# Metabolic vulnerabilities of metastasizing cancer cells

**DOI:** 10.1186/s12915-019-0672-2

**Published:** 2019-07-11

**Authors:** Sarah-Maria Fendt

**Affiliations:** 1 Laboratory of Cellular Metabolism and Metabolic Regulation, VIB-KU Leuven Center for Cancer Biology, VIB, Herestraat 49, 3000 Leuven, Belgium; 2 Laboratory of Cellular Metabolism and Metabolic Regulation, Department of Oncology, KU Leuven and Leuven Cancer Institute (LKI), Herestraat 49, 3000 Leuven, Belgium

## Abstract

Most cancer patients die due to metastasis formation. Therefore, understanding, preventing, and treating metastatic cancers is an unmet need. Recent research indicates that cancer cells that undergo metastasis formation have a distinct metabolism that can be targeted. Here, I would like to discuss potential opportunities in exploiting the metabolic vulnerabilities of metastasizing cancer cells.

## Why is metastasis formation the leading cause of death in cancer patients?

The reason for this is a combination of two major factors. On the one hand, surgical removal becomes challenging when multiple secondary tumors in one or more distant organs arise. On the other hand, many secondary tumors are resistant to targeted therapy; thus, the last line of defense is chemotherapy. Once this fails or the benefit:site effect ratio tilts cancer becomes a terminal disease.

## What are the different steps of metastasis formation?

**Fig. 1.**
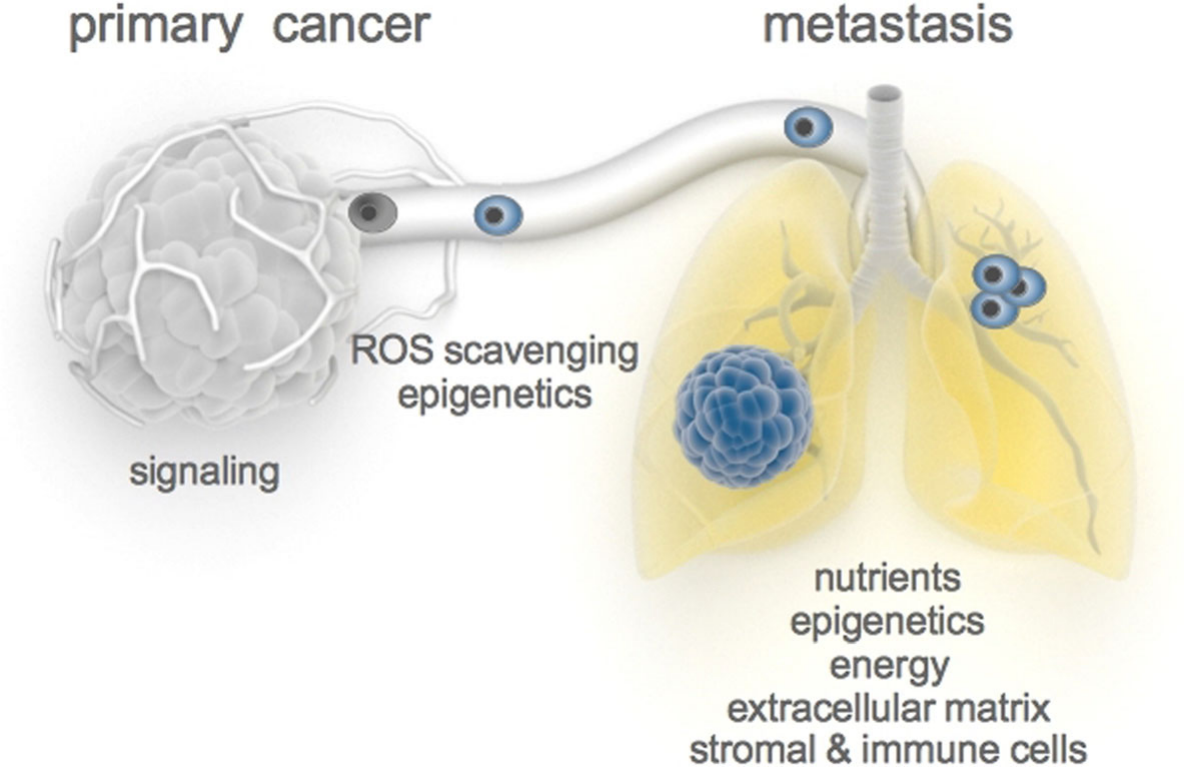
Metastasis formation is a multistep process that requires plasticity in the cancer cell phenotype. This plasticity is at least in part driven by metabolic rewiring and can be impaired by targeting the corresponding metabolic processes. *ROS* reactive oxygen species. Image elements credited to http://www.somersault1824.com

Metastasis formation depends on a cascade of events. First, cancer cells disseminate from the primary tumor and invade into the healthy tissue. Next, they need to survive in the circulation. Once they have reached a distant organ they seed and colonize the new environment. Within this step prolonged periods of dormancy or pseudo-dormancy (balance between cell division and cell death) can occur. The duration of such a (pseudo)dormancy can range from months to years. Yet, some cancer cells might eventually succeed in colonization and transition into a proliferating metastasis, i.e., a secondary tumor (Fig. 1).

## What is the rationale for targeting metabolism during metastasis formation?

Transition through the metatstatic cascade equires that cancer cells change their cellular phenotype between distinct states such as proliferation, invasion, survival, and colonization and this, in turn, requires metabolic changes. In particular, cancer cells require different amounts of metabolic products, including energy, antioxidants, metabolites, and biosynthetic building blocks. Moreover, the nutrients available to cancer cells change dependent on the environment in which they reside. Consequently, cancer cells need to rewire their metabolism in response to the available nutrients and the metabolic products they require to undergo phenotypic changes. Interestingly, the metabolic rewiring allowing cancer cells to transition through the metastatic cascade often differs from the metabolic program of healthy cells. For example, healthy and cancer cells rely on α-ketoglutarate to drive extracellular matrix metabolism. Metastasizing cancer cells require the nutrient pyruvate to drive α-ketoglutarate availability [1], yet non-transformed cells use other nutrients to produce this regulatory metabolite [2]. Thus, the aberrant extracellular matrix metabolism of cancer cells can be normalized by targeting pyruvate uptake and this treatment is expected to have no adverse effects on non-transformed extracellular matrix-producing cells. Therefore, targeting metabolism has the potential to prevent the successful transition of cancer cells through the different steps of metastasis formation without targeting normal cells and healthy tissue.

## How do changes in metabolism support cancer cell invasion?

The invasion of cancer cells into the surrounding tissue is the first step of metastasis formation. This step requires cancer cells to degrade the extracellular matrix, gain motility, and undergo directed migration. These large phenotypic changes are often coordinated by the distinct regulation of protein sets (needed, e.g., for an epithelial-to-mesenchymal transition) and thus require differential activation of signaling pathways. It emerges that many metabolic alterations that have been found to support invasiveness across different tumor types converge into the activation of signaling pathways [3]. As such, metabolic rewiring is upstream of the signaling network and targeting it has the potential to prevent global cellular changes needed for the induction of an invasive phenotype in cancer cells.

## How do changes in metabolism support circulating cancer cells?

Cancer cells in the circulation are exposed to harsh conditions because they lose cell–cell or cell–matrix interaction. To survive this, circulating tumor cells respond by building cell clusters [4] and upregulating their antioxidant metabolism needed for reactive oxygen species scavenging [3]. This includes the upregulation of NADPH recycling to recover glutathione, which is the cellular reactive oxygen species scavenger. In mice, it has been shown that scavenging reactive oxygen species by treatment with *N*-acetylcysteine increased the number of circulating melanoma cells and consequently metastasis formation. Thus, increasing oxidative stress or preventing antioxidant metabolism has the potential to reduce the number of circulating tumors cells, which are the seeds for distant metastases.

## How do changes in metabolism support metastatic colonization?

Only very few cancer cells manage to undergo successful metastatic seeding and subsequent colonization of a distant organ. From a metabolic viewpoint, it emerges that cancer cells which are able to sustain or even increase their energy availability in the less favorable environment of a distant organ can succeed in seeding and colonization [3]. Depending on the metastatic site, cancer cells elevate their energy availability through upregulation of classic ATP-producing pathways such as glycolysis and mitochondrial oxidative metabolism, but also unusual pathways such as proline catabolism [5] and ATP scavenging from the extracellular space [6]. Inhibiting these energy-producing pathways decreases the number of arising metastases in mice [3] and might target already disseminated cancer cells. It is tempting to speculate that this increase in ATP production in cancer cells undergoing metastatic colonizing is required at least in part for the trafficking of cancer cell-produced and modified extracellular matrix, a process that shapes the metastatic niche into a more favorable environment supporting metastatic outgrowth [1].

## Does it matter in which organ a cancer is arising?

In very simplified terms one can say cancer is caused by genetic alterations. However, even when considering a continuous increase of mutations in cancer cells, only looking at the genetic landscape cannot explain cancer as a disease. For example, germline mutations in the metabolic enzyme succinate dehydrogenase are associated with cancer development in certain organs. This suggests that the cancer cell origin and thus the baseline state of the cell that undergoes transformation are important during cancer development. Moreover, data from mouse models suggest that, at least in some cancers, the driver mutations are very similar between the primary tumor and the corresponding metastases [7], yet the epigenetic state, which is inherently linked to metabolism, and thus the active cellular programs can differ dramatically [8]. This, in turn, suggests that environmental factors such as oxygen tension and nutrient availability, but also differences in stromal as well as immune cells, define the (metabolic) properties of arising cancers. As a consequence, cancer treatment selection must consider, besides the genetic makeup of the tumor, additional parameters that are at least in part defined by the organ in which the cancer arises.

## What about reoccurrence of cancers in the primary site?

Cancers that reoccur in the primary site are very frequently resistant to the initial successful treatment. This is often the case because the reoccurring tumors arise from a subpopulation of cancer cells that were able to survive treatment. Defining and understanding which features (including metabolic phenotypes) allow cancer cells to withstand treatment will be important to develop novel strategies against these subpopulations.

## The metastatic cascade is complex—what steps should be targeted in patients?

There is no universally applicable answer to this question because this depends on the tumor type and the organ in which the cancer arises. For instance, being able to reduce the invasiveness of brain tumors before surgical removal is very important to spare as much as possible of normal brain tissue and thus decrease the chance and/or extent of cognitive impairment. For some breast cancers it seems that dissemination of cancer cells is a very early event that can occur before diagnoses [3]. Thus, targeting the later steps of metastasis formation might be more promising to prevent metastatic relapse in such cancers. Moreover, depending on the surgical technique applied, targeting circulating cancer cells upon surgical removal of a primary tumor could decrease the risk of subsequent metastasis formation. In general, more research but also more clinical trials assessing treatments that aim to prevent metastasis formation are required.

## Does it matter what a patient eats?

Epidemiological studies show that certain metabolic states that can be impacted by diet, such as obesity, increase risk and mortality rates in patients with certain types of cancers. Mouse studies suggest that, at least in some cancers, cells with tumor-initiating capacity express CD36 (which is a fatty acid receptor) and that stimulation of CD36-expressing cells with the fatty acid palmitate increased metastasis size and frequency [9]. Moreover, dietary supplementation of antioxidants might not be advantageous in certain cancer patients since mouse studies suggest that at least melanoma cells show increased survival in the circulation when mice are treated with the antioxidant *N*-acetylcysteine. Furthermore, caution is required regarding the interaction of antioxidant supplementation and radiation therapy, as well as some chemotherapies that utilize reactive oxygen species to induce cancer cell death. Finally, it has been shown in mice that the efficacy of PI3 kinase inhibitors (which is a targeted treatment for cancers with PI3K activating mutations) can be increased upon ketogenic diet (low carbohydrate, high fat) feeding [10]. This suggests that at least some cancer treatments respond to diet. Further research and diet-controlled clinical trials are necessary to exploit diets to increase cancer treatment efficacy.

## What’s next?

In terms of research, further characterization and understanding of metabolic rewiring during metastasis formation and the impact of nutrients on cancer in general and metastasis formation in particular are required. Moreover, the cancer-centered view needs to be broadened and include the (metabolic) interaction with stromal and immune cells. Accordingly, it will be interesting to see whether immunotherapy is able to prevent metastasis formation and whether there is a synergistic effect between metabolism-targeting strategies and immunotherapy. In terms of translation to the clinic, a major unmet need are clinical trials that focus on metastasis prevention treatment and clinical trials that are conducted in patient cohorts exposed to defined diets.

## Data Availability

Not applicable.
